# Factors Associated with Prolonged Mechanical Ventilation and 30-Day Mortality in Intubated COVID-19 Patients with Invasive Fungal Infections: A Retrospective Observational Study

**DOI:** 10.3390/tropicalmed10050124

**Published:** 2025-05-06

**Authors:** Hung Manh Than, Thang Van Dao, Truong Van Cao, Tuyen Van Duong, Thach Ngoc Pham, Cap Trung Nguyen, Phu Dinh Vu, Nam Van Le, Binh Nhu Do, Phuong Viet Nguyen, Ha Nhi Vu, Duong Minh Vu

**Affiliations:** 1 Emergency Department, National Hospital for Tropical Diseases, Hanoi 11519, Vietnam; 2 Infectious Department, Faculty of Medicine, University of Medicine and Pharmacy, Vietnam National University, Hanoi 11310, Vietnam; 3 International Ph.D. Program in Medicine, College of Medicine, Taipei Medical University, Taipei 11031, Taiwan; 4 Department of Infectious Diseases, Military Hospital 103, Vietnam Military Medical University, Hanoi 12108, Vietnam; 5 Training and Direction Center, Institute of Military Preventive Medicine, Hanoi 11519, Vietnam; 6 School of Nutrition and Health Sciences, Taipei Medical University, Taipei 11031, Taiwan; 7 Director Office, National Hospital for Tropical Diseases, Hanoi 11519, Vietnam; 8 Intensive Care Unit, National Hospital for Tropical Diseases, Hanoi 11519, Vietnam; 9 Department of Military Science, Vietnam Military Medical University, Hanoi 12108, Vietnam; 10 Department of Microbiology, Faculty of Basic Medicine, Thai Nguyen University of Medicine and Pharmacy, Thai Nguyen 24117, Vietnam; 11 Intensive Care Unit, Military Hospital 103, Vietnam Military Medical University, Hanoi 12108, Vietnam

**Keywords:** COVID-19, invasive fungal infection, prolonged mechanical ventilation, associated factors, mortality risk

## Abstract

COVID-19-associated invasive fungal infections (CAIFIs) contribute to increased mortality and morbidity rates. This study explores the epidemiology, laboratory parameters, radiological characteristics, treatments, and 30-day mortality risks of CAIFI in critically ill intubated patients while also evaluating factors associated with prolonged mechanical ventilation (PMV) in this population. Adults admitted to a tertiary hospital from 1 April 2021 to 31 March 2022 who were diagnosed with severe COVID-19, required invasive mechanical ventilation, and developed invasive fungal infection (IFI) during hospitalization were analyzed in this retrospective cohort study. Among 150 patients, 65 (43.3%) required PMV, with an in-hospital mortality rate of 64%. *Candida albicans* (47%) and *Aspergillus fumigatus* (27%) were the most prevalent pathogens. Multivariate analysis revealed that COVID-19 vaccination (adjusted odds ratio, aOR = 0.155, 95% confidence interval, 95% CI = 0.029–0.835, *p* = 0.030) and higher serum protein levels (aOR = 0.900, 95% CI = 0.819–0.989, *p* = 0.028) were significantly associated with a reduced risk of PMV. Meanwhile, elevated glucose levels (hazard ratio, HR = 1.047, 95% CI = 1.003–1.093, *p* = 0.036) and an increased neutrophil-to-lymphocyte ratio (HR = 1.024, 95% CI = 1.009–1.039, *p* = 0.002) were correlated with a greater 30-day mortality risk. Tracheostomy emerged as a protective factor, significantly reducing the risk of 30-day mortality (HR = 0.273, 95% CI = 0.127–0.589, *p* = 0.001). In this single-center study, patients with CAIFI exhibit a high mortality rate. Clinicians should maintain vigilance for IFI in critically ill COVID-19 patients with mechanical ventilation.

## 1. Introduction

Coronavirus disease 2019 (COVID-19), caused by severe acute respiratory syndrome coronavirus 2 (SARS-CoV-2), is a multi-system disorder associated with significant morbidity and mortality, primarily due to progressive life-threatening pneumonia [1]. The ongoing COVID-19 pandemic continues to place substantial strain on global healthcare systems. As of 15 September 2024, over 776 million infections had been reported, with more than 7 million confirmed deaths worldwide [2]. Although the mortality rate of severe COVID-19 patients admitted to intensive care units (ICUs) has been declining since the emergence of the Omicron variant, critically ill patients requiring invasive mechanical ventilation (IMV) continue to exhibit high mortality rates [3,4]. Among these patients, invasive fungal infection (IFI) has become one of the most prevalent secondary infections [5]. Recorded incidences of COVID-19-associated invasive fungal infections (CAIFIs) vary widely, ranging from 5% to 30% in severe populations [6,7,8]. Compared with non-COVID-19 patients, the incidence of candidemia has been reported at two- to tenfold higher frequency in patients with COVID-19 [9,10].

Recent investigations suggested that standard-of-care corticosteroids for COVID-19 treatment may contribute to an increased risk of IFI [11]. Other factors associated with heightened CAIFI risk have been identified, including pre-existing comorbidities, prior antibiotic exposure, and prolonged mechanical ventilation (PMV) [12]. Disease-specific intrinsic factors, such as immune dysregulation [13], lung parenchymal damage, reduced lung compliance [14], increased thrombosis risk [15], and alterations in the lung microbiome [16], also play a role in creating conditions conducive to secondary infections. Previous studies indicated that CAIFI significantly increases mortality during the pandemic, particularly in mechanically ventilated patients [8]. A United Kingdom cohort reported a 55% mortality rate among patients with probable COVID-19-associated pulmonary aspergillosis (CAPA), nearly doubling the 90-day mortality risk [17]. Crude mortality rates associated with *Candida* spp. infections range between 30% and 80% [18], whereas pulmonary or disseminated mucormycosis exceeds 80% [19]. These findings underscore the substantial burden of IFIs on critically ill COVID-19 patients.

Prolonged mechanical ventilation (PMV) is a recognized risk factor for developing IFIs, but it can also be a consequence of these infections [20]. PMV has been broadly associated with increased healthcare resource utilization and adverse clinical outcomes, including elevated mortality rates. A meta-analysis of 29 studies on PMV revealed a pooled one-year mortality rate of 62% [21]. Numerous studies have identified risk factors for PMV in non-COVID-19 patients, including comorbidities, sepsis, multi-drug resistant infection, and malnutrition [20,21]. However, little has been published on the prognostic factors for requiring PMV in COVID-19 patients [22], especially those with IFI. Additionally, the identification of risk factors for mortality in this population remains insufficiently documented, particularly in low- and middle-income countries (LMICs).

Understanding risk factors for PMV and mortality is crucial for clinicians to classify and prognosticate patients early, thereby providing early and prompt therapy to improve survival rates in patients with CAIFI. Therefore, we conducted this study to investigate the epidemiology, laboratory profile, radiologic findings, therapies, and 30-day mortality risks of CAIFI in critically ill intubated patients. Moreover, factors associated with PMV in these patients were assessed in this study.

## 2. Materials and Methods

### 2.1. Study Design and Population

This retrospective observational study was conducted at the National Hospital for Tropical Diseases (NHTD), one of the largest tertiary hospitals in Vietnam specializing in the treatment of tropical diseases, including severe COVID-19 cases during the pandemic, located in the Hanoi capital.

The eligible patients were adults (age > 18 years) admitted to the Emergency Department and Intensive Care Unit (ICU) of NHTD between 1 April 2021 and 31 March 2022, who were diagnosed with severe COVID-19, required IMV, and developed IFI during hospitalization. Admission to the Emergency Department and ICU and treatment decisions were left to the discretion of the attending physicians following national guidelines [23,24]. Patients were followed from admission until death, discharge, or transfer to another healthcare facility.

### 2.2. Data Collection

A retrospective review was performed for all the eligible patients. This study focused on all-cause mortality. Data were extracted from electronic medical records, including demographic information (age, sex), body mass index (BMI), comorbidities (diabetes, chronic kidney disease CKD, hypertension, history of stroke, chronic obstructive pulmonary disease COPD, malignancy), sequential organ failure assessment (SOFA) score, and COVID-19 vaccination status.

Laboratory profiles were collected when patients were diagnosed with IFI. The study was also interested in the neutrophil-to-lymphocyte ratio (NLR) and lymphocyte-to-C-reactive protein ratio (LCR). Data for interventions and medications were gathered, including details on tracheostomy, extracorporeal membrane oxygenation (ECMO), continuous renal replacement therapy (CRRT), corticosteroid treatment, antifungal treatment, and antiviral therapy. In addition, radiological results that were carried out during hospitalization were collected.

Microbiological data regarding fungi isolated from endotracheal aspirate (ETA) and blood specimens were compiled, with results of multiple samples taken from patients throughout the patients’ hospital stays. Fungal strains were isolated and identified according to the standard procedures of the Ministry of Health and the manufacturer [25]. Patient outcomes, defined as death or survival, were recorded based on the discharge status.

### 2.3. Detection of Fungi

Initially, the specimens were prepared and cultured on Sabouraud dextrose agar (SDA) with chloramphenicol and with/without cycloheximide, using duplicate plates. The cultures were incubated at 30 °C and room temperature. Then, fungal growth was evaluated based on microscopic examination and colony morphology to identify fungal structures.

In cases of yeast growth, colonies were aseptically transferred to fresh Chromogenic candida agar (CCA) or SDA plates to obtain pure cultures. Yeast identification was performed using the automated VITEK^®^ 2-COMPACT system, following the standard yeast identification protocol.

For filamentous fungi identified as *Aspergillus*, isolates were subcultured onto a Czapek medium designed for *Aspergillus* to enable the identification of pathogenic species.

### 2.4. Definitions

Patients were confirmed with COVID-19 by detecting SARS-CoV-2 RNA via reverse-transcription polymerase chain reaction (RT-PCR) testing performed on nasopharyngeal samples. COVID-19 cases were classified as severe according to the guidelines for diagnosis and treatment of COVID-19 issued by the Ministry of Health at that time [23,24]. PMV was determined as IMV from the date of IFI diagnosis lasting >17 days, a threshold commonly used in prior reports to reflect real-world COVID-specific data [22,26].

In this study, COVID-19 vaccination status was ascertained based on information provided by patients or their relatives regarding receipt of any COVID-19 vaccine prior to hospitalization. Individuals who had received at least one dose, irrespective of the vaccine type or the timing of administration, were classified as vaccinated. Those who had not received any COVID-19 vaccine were categorized as unvaccinated.

IFIs were diagnosed, adhering to the EORTC/MSGERC criteria for ICU patients [27]. Invasive candidiasis (Candidemia) was determined by the detection of *Candida* species in sterile body fluid (blood). Probable CAPA was identified based on mycological evidence of *Aspergillus* spp. detected in a lower respiratory tract sample through direct microscopy and culture, combined with clinical and radiological abnormalities [27]. The date of IFI diagnosis was defined as the initiation date of antifungal therapy.

### 2.5. Outcomes

Mortality occurring within 30 days following the diagnosis of IFI was determined as 30-day crude mortality. PMV was also a primary outcome of this study. Risk factors associated with 30-day mortality and PMV were analyzed using independent clinical parameters.

### 2.6. Statistical Analysis

The descriptive analysis of independent parameters was compared between the prolonged and short mechanical ventilation groups. Categorical parameters were summarized using frequencies and percentages, while continuous parameters were reported as medians with interquartile ranges (IQR: 25th–75th percentile) or means and standard deviations, depending on the data distribution. Student’s *t*-test was applied to compare means of normally distributed continuous variables, whereas the Mann–Whitney U test was used for evaluating medians of non-normally distributed data. Categorical data were assessed using either the Chi-square test or Fisher’s exact test based on the suitability of the dataset.

Logistic regression analysis was conducted to identify factors associated with PMV, and Cox proportional hazard regression was utilized to determine predictors of 30-day mortality. Data for demography, BMI, comorbidity, SOFA scores, COVID-19 vaccination status, laboratory tests, microbiology, and treatments were tested in the univariate analysis. Parameters with a *p* < 0.05 in the univariate analysis were considered for inclusion in the multiple regression analysis. A correlation matrix was utilized to check multicollinearity among independent continuous variables before conducting a multiple regression model. When a high correlation between variables was found (∣ρ∣ > 0.6), one of the correlated variables was selected to reduce redundancy. Factors associated with PMV were expressed as an odds ratio (OR) with 95% confidence interval (CI), while those linked to 30-day mortality were presented as a hazard ratio (HR) with 95% CI. Statistical significance was set at *p* < 0.05. All statistical computations were performed using SPSS 22.0 (SPSS software, Armonk, NY, USA) and Microsoft Excel 365 (Microsoft Corp., Redmond, WA, USA).

## 3. Results

**Figure 1:**
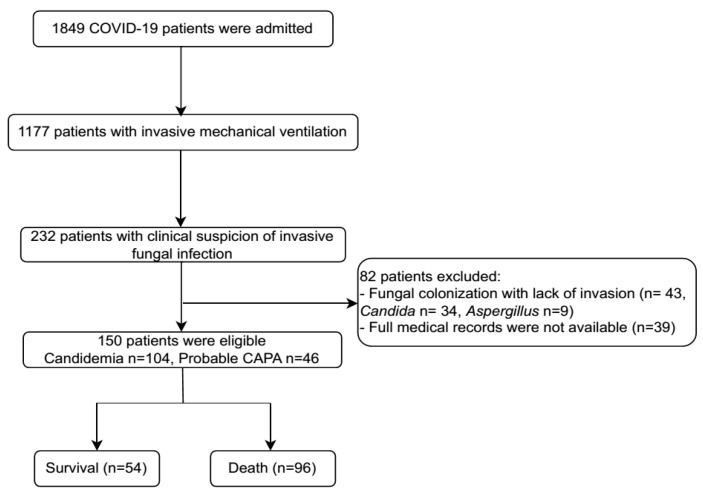
Patient flow chart. CAPA: COVID-19-associated pulmonary aspergillosis.

From 1 April 2021 to 31 March 2022, 1849 individuals with confirmed COVID-19 were admitted to the Emergency Department and ICU at NHTD. Among these patients, 1177 presented with severe illness requiring IMV. Within this cohort, 232 patients with clinical suspicion of IFI were identified. However, 82 patients were excluded due to fungal colonization (*Candida/Aspergillus*) in respiratory samples without evidence of invasion or incomplete medical records. Eventually, 150 patients were included in the final analysis, comprising 104 cases of candidemia and 46 cases of probable CAPA (Figure 1).

### 3.1. Characteristics of the Study Patients and Outcomes

Of the 150 eligible patients, 65 (43.3%) required PMV, and 72 (48%) were female. The median age was 67 (IQR: 58–76) years. The most frequent comorbidities were hypertension (57.3%) and diabetes (24.7%). No significant differences were observed between the two groups in diabetes, CKD, hypertension, COPD, and malignancy except for a history of stroke (*p* = 0.008). Obesity in 47.3% of patients was significantly more frequent in the PMV group (*p* = 0.021). The percentage of individuals with prior COVID-19 vaccination was 25.3%, significantly lower in the PMV group, *p* = 0.014. The median SOFA score was 4 (IQR: 2–5). The median duration from admission to the diagnosis of IFI was 9 days (IQR: 7–12). At the time of IFI diagnosis, the median PaO_2_/FiO_2_ ratio was 112.5 (IQR: 81.3–156.8) (Table 1).

The median length of hospitalization was 21 days (IQR: 14–30), significantly longer in the PMV group (*p* < 0.001). The 30-day crude mortality rate was 52% (78/150), while the in-hospital death rate was 64% (96/150) (Table 1).

### 3.2. Laboratory Parameters on the Day of IFI Diagnosis of the Study Patients

Regarding laboratory parameters at IFI diagnosis of the patients, the median percentage of neutrophils (87.5% vs. 90.4%, *p* = 0.04), CRP levels (64.9 vs. 89.9, *p* = 0.011), and INR (1.15 vs. 1.24, *p* = 0.029) were significantly lower in the PMV than those of the short MV group. The mean protein level was also significantly lower in the PMV group (59.73 vs. 64.22, *p* = 0.029). Meanwhile, the mean value of PT (%) was significantly higher in the PMV group than in the counterpart group (79.2 vs. 73.3, *p* = 0.026). Similarly, the median lymphocyte-to-CRP ratio (LCR) was significantly elevated in the PMV group (0.98 vs. 0.56, *p* = 0.008) (Table 2).

Other hematological parameters, such as WBC count, lymphocytes (%), platelets, and hemoglobin, illustrated no significant differences between the groups. Kidney function markers (urea, creatinine), glucose, liver enzymes (AST, ALT), and electrolyte parameters (sodium, potassium, chloride) also exhibited no significant differences. D-dimer levels tended to be higher in the PMV. The two groups had no statistically significant differences in albumin, procalcitonin, APTT, fibrinogen, and NLR (Table 2). Laboratory parameters on the IFI diagnosis day of the study patients according to mortality were demonstrated in Supplementary Table S1.

**Figure 2:**
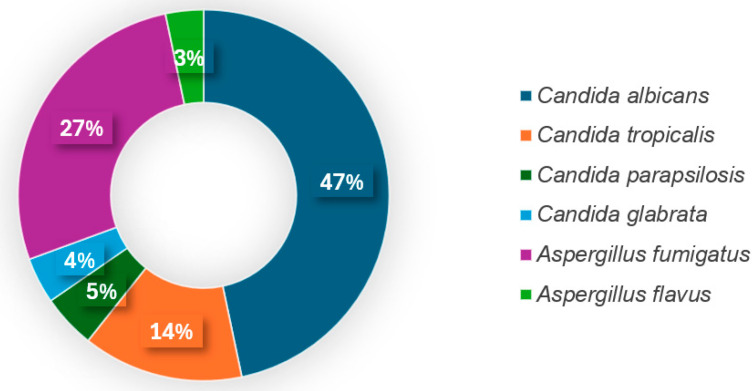
Fungal pathogens causing IFI in intubated COVID-19 patients.

Among the detected fungi, *Candida albicans* (47%, 70/150) and *Aspergillus fumigatus* (27%, 41/150) were the most prevalent. *Candida tropicalis* was identified in 14% (21/150) of samples, while *Candida parapsilosis* (5%, 7/150), *Candida glabrata* (4%, 6/150), and *Aspergillus flavus* (3%, 5/150) were less frequently detected (Figure 2).

### 3.3. Radiologic Findings, Interventions and Medications

A CT scan was conducted in 79 patients (52.7%). In the 46 cases of probable CAPA, the most common imaging lesion was ground glass opacities, accounting for 93.5% (43/46), followed by consolidations (35/46, 76.1%). A nodule was witnessed in 12 cases (26.1%), and bronchial wall thickening was detected in 10 cases (21.7%) (Supplementary Table S2).

With regard to interventions, the rates of patients who received tracheostomy, ECMO, and CRRT were 53.3%, 8%, and 60.7%, respectively. Patients with PMV received tracheostomy, ECMO, and CRRT significantly more often (*p* < 0.001). In addition, 99.3% (149) of patients received corticosteroid therapy, and the majority of patients (90%) were treated with dexamethasone. The median duration of corticoid treatment was 11 days (IQR: 8–16), significantly longer in the PMV group (*p* = 0.002). Among 150 patients, 66.7% (100 patients) did not receive antiviral therapy. Of the remaining, 49 patients (32.6%) were treated with remdesivir, and one patient (0.7%) received favipiravir (Table 1).

Regarding antifungal treatment, 122/150 patients (81.3%) received antifungal treatment, with a median therapy duration of 15 days (IQR: 8–29). A total of 54 patients (36%) were initiated on fluconazole, 37 (24.7%) on voriconazole, 25 (16.7%) on echinocandins, 5 patients (3.2%) on itraconazole, and 1 patient (0.7%) on amphotericin B. Initial antifungal treatment was adjusted in 51 patients, switching to another antifungal class. Specifically, 16 patients were switched to an echinocandin (5 patients initially on fluconazole, 11 patients initially on voriconazole), while 21 patients were switched to voriconazole (8 patients initially on fluconazole, 11 patients on echinocandins, and 2 patients on itraconazole). Fluconazole became the second-line therapy for 11 patients (6 patients initially on echinocandin and 5 people on voriconazole). Additionally, one patient was transitioned from amphotericin B to itraconazole, and two patients were switched to amphotericin B from fluconazole. In this study, 28 patients (18.7%) did not receive antifungal treatment (Supplementary Table S3).

### 3.4. Factors Associated with PMV

The univariate logistic regression analysis results identifying factors associated with PMV are provided in Supplementary Table S4 and Table 3. The analysis indicated that antifungal treatment was not significantly associated with a decreased risk of PMV (OR = 0.558, 95%CI = 0.234–1.332, *p* = 0.189). Also, antiviral treatment did not demonstrate a significant association with a reduced risk of PMV (OR = 0.340, 95%CI = 0.162–1.117, *p* = 0.056) (Supplementary Table S4). In contrast, a longer duration of corticosteroid treatment (OR = 1.049, 95%CI:1.011–1.088, *p* = 0.011) was significantly associated with an increased risk of PMV. However, this contribution was witnessed only in the univariate logistic regression model (Table 3).

In the multivariate logistic analysis, COVID-19 vaccination and serum protein levels were significant predictors of PMV. In particular, COVID-19 vaccination was strongly associated with a reduced risk of PMV (aOR = 0.155, 95%CI =0.029–0.835, *p* = 0.03). Likewise, higher protein levels were linked to a lower likelihood of PMV (aOR = 0.90, 95%CI = 0.819–0.989, *p* = 0.028) (Table 3).

### 3.5. Risk Factors for 30-Day Mortality

The simple Cox regression analysis was initially performed to investigate 30-day mortality risk in the study patients (Supplementary Table S5 and Table 4). The analysis revealed that neither antifungal nor antiviral therapies were significantly associated with a decreased risk of 30-day mortality (HR = 0.859, 95%CI= 0.482–1.533, *p* = 0.607 for antifungal therapy, HR = 0.588, 95%CI = 0.370–1.934, *p* = 0.250 for antiviral treatment). The duration of corticosteroid treatment was also not associated with 30-day mortality risk (HR = 0.965, 95%CI:0.940–1.990, *p* = 0.07) (Supplementary Table S5). Prior COVID-19 vaccination significantly reduced the risk of mortality (HR = 0.509, 95%CI = 0.315–0.822, *p* = 0.006). However, these associations were not observed in the multiple regression model (Table 4).

The results of the multicollinearity examination among continuous parameters chosen from the simple Cox regression analysis are provided in Supplementary Table S6. Multivariate risk factor analysis showed that glucose level, NLR, and tracheostomy were significant predictors of 30-day mortality in patients with CAIFI. Particularly, elevated glucose levels were associated with a higher risk of mortality (HR = 1.047, 95%CI = 1.003–1.093, *p* = 0.036). Similarly, an increased neutrophil-to-lymphocyte ratio was correlated with a greater mortality risk (HR = 1.024, 95%CI = 1.009–1.039, *p* = 0.002). Conversely, tracheostomy was evaluated as a protective factor, significantly reducing the risk of 30-day mortality in these patients (HR = 0.273, 95%CI = 0.127–0.589, *p* = 0.001). Another notable finding was that patients with prior COVID-19 vaccination tended to have a lower risk of 30-day mortality (HR = 0.475, 95%CI = 0.219–1.029, *p* = 0.059) (Table 4).

## 4. Discussion

To the best of our knowledge, this is the first study in Vietnam investigating risk factors for PMV and mortality in intubated COVID-19 patients with IFI. In our study, the in-hospital mortality rate was 64% among patients with CAIFI. Regarding PMV outcome, COVID-19 vaccination and higher protein levels were identified as protective factors. Higher levels of glucose and elevated NLR were significantly associated with an increased risk of 30-day mortality, while tracheostomy was found to reduce the risk of 30-day mortality in these patients.

The high in-hospital mortality rate (64%) observed in critically ill intubated patients with CAIFI in this study is in line with findings from a meta-analysis by Hoenigl et al. (2022), which reported mortality rates ranging from 47 to 80% in CAPA and other fungal infections, particularly in mechanically ventilated patients [10]. Similarly, a multicenter study conducted in France found an ICU mortality rate of 61.8% among patients with proven or probable CAPA [8]. A meta-analysis estimated that the fatality rate of patients with Coronavirus-associated *C. auris* infection (CACa) was around 68% [28]. For mucormycosis, mortality rates have been reported to exceed 80% [19]. In our study, over half of patients (52%) were reported to have died within 30 days after IFI diagnosis. The variations in mortality rates across studies can be attributed to factors such as the quality of care, the incidence of ICU-acquired infections, admission and discharge criteria, ICU type, equipment utilization, and staff workload, including the nurse-to-patient ratio. Nevertheless, it is important to note that the mortality rate among patients with CAIFI remains high across ICUs, irrespective of whether they are located in developed or developing countries.

In the present study, the pathogens identified among patients with CAIFI were *Aspergillus* and *Candida*. In particular, *Candida albicans* was the most frequently isolated fungus, accounting for 47% (70/150) of cases, followed by *Aspergillus fumigatus* at 27% (41/150). This distribution aligns with global observations, where *Candida* and *Aspergillus* are predominant pathogens for IFI in critically ill COVID-19 patients undergoing mechanical ventilation. Other fungi, such as *Cryptococcus*, *Coccidioides*, *Histoplasma*, and *Blastomyces*, have been reported to cause CAIFI among severe intubated patients in the United States [29]. Additionally, COVID-19-associated mucormycosis (CAM) has emerged as a significant complication, particularly in regions like India, where a notable increase in cases has been documented, with estimates indicating a 50-fold increase compared to pre-pandemic levels [10,30]. Contrary to these reports, no such cases were identified in our cohort. In Vietnam, mucormycosis remains rare, with an annual incidence of approximately 0.2 cases per 100,000 population, and no notable increase has been linked to the COVID-19 pandemic [31].

In the NHTD, a culture-based methodology was employed to identify the causative fungal pathogens involved. Traditional culture techniques are considered the gold standard for IFI diagnosis, as they enable precise species identification, which is essential for clinicians to select appropriate antifungal therapies, thereby preventing the development of resistance, reducing treatment failures, and ensuring effective patient management. Moreover, accurate species identification assists in distinguishing between colonization and true infection, which helps avoid unnecessary antifungal treatments, thus minimizing potential side effects and healthcare costs. However, culture-based diagnostic methods for fungal infections face several limitations, including low sensitivity and labor-intensive procedures [32,33]. In addition, numerous cryptic fungi cannot be cultivated or isolated using standard culture media, rendering them undetectable through conventional techniques. A particularly significant disadvantage of culture methods is their prolonged turnaround time compared to molecular techniques (e.g., PCR, DNA sequencing) or serological tests (e.g., Galactomannan, β-D-Glucan), which can hinder timely treatment planning and diminish the effectiveness of antifungal therapy [33,34]. Our findings indicated no significant difference in outcomes, including PMV (OR = 0.558, *p* = 0.189) and 30-day mortality (HR = 0.859, *p* = 0.607), between patients who received antifungal treatment and those who did not. Prior studies have emphasized that the early recognition and treatment of IFIs are vital to prevent life-threatening complications and reduce mortality rates [35,36]. Therefore, while microbiological and histopathological diagnostic tools are essential for definitive diagnoses, there is a pressing need to develop novel fungal identification methods that are rapid, cost-effective, and highly specific. Such advancements would be particularly beneficial for resource-limited settings in LMICs, facilitating earlier diagnosis and improving patient outcomes.

Previous studies have demonstrated that COVID-19 vaccination significantly decreases the risk of severe COVID-19, ICU admissions, mechanical ventilation, and mortality [37,38,39]. An Italian cohort study reported an ICU admission incidence rate ratio (IRR) of 0.03 for vaccinated individuals [37]. Another study found that vaccination lowered in-hospital mortality and IMV risk, with aORs of 0.42 and 0.40, respectively [39]. In Vietnam, during the study period, at least eight COVID-19 vaccines were granted for Emergency Use Authorization, including AstraZeneca, Pfizer-BioNTech, Moderna, Johnson & Johnson, Sinopharm, Sputnik V, Hayat-Vax, and Covaxin. Among these, the most widely administered vaccines were AstraZeneca, Pfizer-BioNTech, and Sinopharm [40]. In addition, a government-led vaccination campaign raised full vaccination coverage from 24.7% in September 2021 to 86.9% by December 2022, lowering mortality from 2.5% to ~0.4% [40,41]. However, the direct impact of vaccination on the incidence of PMV in COVID-19 patients remains underexplored. In our study, 25.3% of the patients had received COVID-19 vaccination, which was associated with a significant reduction in the risk of PMV (aOR = 0.155, *p* = 0.030). Furthermore, COVID-19 vaccination was identified as a protective factor for 30-day mortality in the simple regression model (HR = 0.509, *p* = 0.006) and in the multivariate regression analysis (HR = 0.475, *p* = 0.059). Our findings underline the critical role of COVID-19 vaccination in mitigating mortality, especially in preventing the need for PMV among intubated patients with CAIFI.

In recent years, the NLR has emerged as a valuable marker of immune dysregulation and inflammatory response. In COVID-19 patients, an elevated NLR is associated with more respiratory failure, prolonged ICU stays, and increased mortality [42]. Consistent with these findings, our study discovered NLR as an independent predictor of 30-day mortality in intubated COVID-19 patients with IFI (HR = 1.024, *p* = 0.002). The increased NLR in severe COVID-19 patients is driven by the overactivation of neutrophils due to systemic inflammation and immune hyperactivation, combined with lymphocyte depletion resulting from viral effects, immune exhaustion, and apoptosis [43,44]. NLR offers greater sensitivity and specificity compared to white blood cell count (WBC), an earlier rise in response, and a longer persistence, making it a reliable tool for identifying patients with severe disease [45,46]. Additionally, given its simplicity and prognostic value, NLR serves as a significant biomarker to guide clinical management, particularly in resource-constrained settings.

Interestingly, in our study, 60.7% of COVID-19 patients required CRRT, disclosing highly frequent acute kidney injury (AKI) in this population. Comparatively, a study in Korea found that only 19.2% (123/640) of mechanically ventilated COVID-19 patients required CRRT [47], while another study estimated CRRT use in critically ill COVID-19 patients at up to 35% [48]. Fisher et al. (2020) reported an AKI incidence of 87.2% in COVID-19 ICU patients versus 65% in non-COVID ICU cases [49]. The AKI in COVID-19 is influenced by complex mechanisms [50,51,52]. Direct viral injury plays a critical role, as SARS-CoV-2 infects renal cells through Angiotensin-Converting Enzyme 2 (ACE2) receptors expressed in the kidneys [50]. Additionally, indirect mechanisms contribute to kidney damage, including respiratory infections and inflammation affecting renal vasculature, cytokine storms exacerbating renal dysfunction, increased thrombus formation impairing renal blood flow, and the presence of comorbidities that heighten susceptibility to kidney damage in the context of COVID-19 [51,52]. Several risk factors for AKI in COVID-19 patients have been identified in previous research, comprising patients with the following characteristics: older, male, comorbidities (CKD, hypertension), the use of vasopressors, and mechanical ventilation [53,54]. In addition to these factors, we propose that the impact of IFIs and antifungal treatments on the progression of AKI in COVID-19 patients warrants further investigation.

Regarding tracheostomy, this intervention was performed in approximately 8–13% of patients requiring advanced respiratory support in ICUs in the prior COVID-19 pandemic period [55]. However, during the pandemic, recorded data on tracheostomy use varied considerably, ranging from 16% to 61%, representing a substantial increase compared to pre-pandemic levels [56,57]. In our study, 53.3% of patients underwent tracheostomy, and nearly 90% of patients with PMV received this therapy. Importantly, we found that tracheostomy was associated with a reduced risk of 30-day mortality in COVID-19 patients with IFI (HR = 0.273, *p* = 0.001). Our results provide evidence to inform clinical protocols, highlighting the importance of considering earlier implementation of this intervention for critically ill intubated patients with CAIFI. The potential advantages, containing lower sedation requirements and improved airway management, contributing to shorter ICU stays and improved clinical outcomes, align with findings from a prior meta-analysis [58].

Our study has several limitations. First, a main limitation of this study was the inability to perform diagnostic bronchoscopy on the patients to assess fungal invasiveness appropriately as the procedure poses a risk of cross-contamination and has the potential to further compromise the condition of patients already experiencing respiratory failure, making it difficult to perform safely in this population. Moreover, the lesions on CT findings in patients with probable CAPA may overlap with those caused by COVID-19 pneumonia, bacterial superinfections, or other fungal or viral infections, raising the possibility of including cases of *Aspergillus* colonization, which could influence the study’s results. Second, the data were gathered during the early phase of the COVID-19 pandemic, characterized by substantial resource limitations and rapidly changing clinical protocols, which may have contributed to difficulties and variability in diagnosis and treatments. Third, the relatively small sample size, drawn from a single hospital, may limit the robustness and generalizability of the results. Fourth, although prior COVID-19 vaccination was identified as a protective factor for PMV, the study did not provide detailed information on the type, number, or timing of vaccine doses, potentially influencing the interpretation of this finding. Finally, the analysis may not fully apply to the currently dominant viral strains. Within the timeframe of the study, the Delta variant dominated Vietnam from late April 2021 and was characterized by high transmissibility and severity. Then, Omicron emerged in late December 2021 and rapidly replaced Delta to become dominant by early 2022 [59]. Nevertheless, we believe these results provide valuable insights and could serve as a helpful reference for managing future viral pandemics associated with respiratory complications.

## 5. Conclusions

The mortality rate among intubated patients with CAIFI is notably high. Prior COVID-19 vaccination and higher serum protein levels reduce the risk of PMV. Tracheostomy and COVID-19 vaccination decrease 30-day mortality risk, while elevated glucose levels and higher neutrophil-to-lymphocyte ratio are associated with increased risk of 30-day mortality. Prospective studies and multicenter trials should be conducted in the future to validate the findings as well as develop new diagnostic tests and targeted interventions for IFI in intubated COVID-19 patients.

## Figures and Tables

**Table 1:** Characteristics of the study patients, treatments, and outcomes.

Parameter	Total (*n* = 150)	Length of MV		*p*-Value
		PMV (*n* = 65)	Short MV (*n* = 85)	
Age (years)	67 (58–76)	66 (59–73)	67 (57–77)	0.341
Gender				0.553
Male	78 (52)	32 (49.2)	46 (54.1)	
Female	72 (48)	33 (50.8)	39 (45.9)	
Comorbidity				
Diabetes	37 (24.7)	13 (20)	24 (28.2)	0.246
CKD *	13 (8.7)	3 (4.6)	10 (11.8)	0.151
Hypertension	86 (57.3)	32 (49.2)	54 (63.5)	0.096
History of stroke *	17 (11.3)	2 (3.1)	15 (17.6)	0.008
COPD *	7 (4.7)	3 (4.6)	4 (4.7)	0.979
Malignancy *	8 (5.3)	4 (6.2)	4 (4.7)	0.727
BMI				0.021
Non-obesity	79 (52.7)	27 (41.5)	52 (61.2)	
Obesity	71 (47.3)	38 (58.5)	33 (38.8)	
Prior COVID-19 vaccination	38 (25.3)	10 (15.4)	28 (32.9)	0.014
SOFA score at IFI diagnosis	4 (2–5)	4 (2–5)	3 (2–5)	0.413
Time to IFI diagnosis ^#^, days	9 (7–12)	9 (7–13)	8 (7–11)	0.177
PaO_2_/FiO_2_ at IFI diagnosis	112.5 (81.3–156.8)	100.0 (75.5–139.5)	117.0 (89.3–170.8)	0.136
IFI diagnosis				0.077
Candidemia	104 (69.3)	40 (61.5)	64 (75.3)	
Probable CAPA	46 (30.7)	25 (38.5)	21 (24.7)	
Therapeutic interventions				
Tracheostomy	80 (53.3)	57 (87.7)	23 (27.1)	<0.001
ECMO *	12 (8)	11 (16.9)	1 (1.2)	<0.001
CRRT	91 (60.7)	51 (78.5)	40 (47.1)	<0.001
Corticoid treatment (days)	11 (8–16)	16 (9–23)	10 (8–14)	0.002
Type of corticosteroid *				0.860
Dexamethasone	135 (90)	58 (89.2)	77 (90.6)	
Methylprednisolone	13 (8.6)	6 (9.2)	7 (8.2)	
Hydrocortisone	1 (0.7)	1 (1.6)	0 (0)	
No corticosteroid	1 (0.7)	0 (0)	1 (1.2)	
Antiviral medications *				0.045
No antiviral treatment	100 (66.7)	51 (78.5)	49 (56.6)	
Remdesivir	49 (32.6)	13 (20)	36 (42.4)	
Favipiravir	1 (0.7)	1 (1.5)	0 (0)	
Outcomes				
Hospital LOS (days)	21 (14–30)	31 (23–44)	15 (10–20)	<0.001
30-day mortality	78 (52.0)	30 (46.2)	48 (56.5)	0.089
14-day mortality	43 (28.7)	17 (26.2)	26 (30.6)	0.498
In-hospital mortality	96 (64.0)	40 (61.5)	56 (65.9)	0.583

Data are expressed as *n* (%) or median and interquartile range (IQR). * Results were run by Fisher’s exact test. Abbreviations: MV, mechanical ventilation; PMV, prolonged mechanical ventilation; CKD, chronic kidney disease; COPD, chronic obstructive pulmonary disease; BMI, body mass index; SOFA, sequential organ failure assessment; PaO_2_/FiO_2,_ partial pressure of oxygen in arterial blood-to-fraction of inspired oxygen ratio; ECMO, extracorporeal membrane oxygenation; CRRT, continuous renal replacement therapy; LOS, length of stay. ^#^ Time to IFI diagnosis is determined as the interval between the dates of admission and IFI diagnosis.

**Table 2:** Laboratory parameters on the day of IFI diagnosis of the study patients.

Parameter	Total (*n* = 150)	Length of MV		*p*-Value
		PMV (*n* = 65)	Short MV (*n* = 85)	
WBC (×10^9^/L)	10.8 (7.1–14.5)	10.7 (6.6–13.2)	15.0 (11.3–19.1)	0.195
Neutrophils (%)	89.4 (84.1–92.9)	87.5 (83.3–91.3)	90.4 (84.8–93.7)	0.040
Lymphocytes (%)	5.95 (2.98–9.73)	7.00 (3.50–10.00)	5.20 (2.70–8.50)	0.122
Hemoglobin (g/L)	122.0 (108.8–139.0)	122.0 (105.5–139.5)	122.0 (111.0–138.0)	0.962
Platelets (×10^9^/L)	186.5 (149.0–239.8)	181.0 (140.0–238.5)	195.0 (153.5–246.0)	0.247
Urea (mmol/L)	6.80 (4.95–11.60)	6.65 (4.98–10.68)	7.50 (4.98–12.0)	0.446
Creatinine (µmol/L)	79.9 (60.6–104.5)	77.0 (57.3–95.5)	81.0 (61.4–110.1)	0.554
Glucose (mmol/L)	9.80 (7.45–13.70)	9.20 (6.35–11.80)	10.30 (7.55–15.28)	0.220
Protein (g/L)	62.27 (8.0)	59.73 (6.03)	64.22 (8.77)	0.029
Albumin (g/L)	30.12 (5.68)	29.71 (5.48)	30.43 (5.84)	0.481
AST (UI/L)	48.0 (34.1–78.7)	46.0 (31.8–68.8)	54.0 (34.5–81.1)	0.120
ALT (UI/L)	34.3 (24.8–51.1)	34.0 (20.8–51.4)	34.3 (23.0–51.0)	0.997
CRP (mg/L)	80.2 (44.0–127.6)	64.9 (32.9–112.0)	89.9 (55.7–141.7)	0.011
Sodium (mmol/L)	137.0 (133.4–140.0)	136.6 (134.8–140.0)	137.0 (133.0–140.5)	0.678
Potassium (mmol/L)	3.93 (3.60–4.30)	3.90 (3.60–4.25)	3.96 (3.60–4.37)	0.817
Chloride (mmol/L)	101.4 (97.8–105.7)	101.5 (98.4–105.5)	101.3 (26.8–106.0)	0.607
Procalcitonin (ng/mL)	0.32 (0.14–0.95)	0.30 (0.14–0.87)	0.48 (0.13–1.14)	0.530
D-Dimer (ng/L)	1287.0 (728.0–3849.0)	1307.0 (639.0–4633.3)	1287.0 (766.0–3727.0)	0.921
PT (%)	75.8 (16.0)	79.2 (15.9)	73.3 (15.7)	0.026
APTT (s)	32.7 (29.3–37.4)	32.9 (29.4–37.8)	32.3 (29.0–36.5)	0.614
INR	1.21 (1.11–1.32)	1.15 (1.09–1.27)	1.24 (1.12–1.33)	0.029
Fibrinogen (g/L)	4.56 (1.15)	4.42 (1.10)	4.67 (1.18)	0.223
NLR	15.16 (8.85–30.59)	12.77 (8.47–26.46)	17.14 (10.31–34.79)	0.112
LCR	0.65 (0.36–1.63)	0.98 (0.40–2.07)	0.56 (0.34–1.08)	0.008

Normally distributed continuous data were demonstrated as mean and standard deviation (SD). Skewed continuous data were demonstrated as median and interquartile range (IQR). Abbreviations: WBC, white blood cells; AST, aspartate aminotransferase; ALT, alanine aminotransferase; CRP, C-reactive protein; PT, prothrombin time; APTT, activated partial thromboplastin time; INR, international normalized ratio; NLR, neutrophil-to-lymphocyte ratio; LCR, lymphocyte-to-C-reactive protein ratio.

**Table 3:** Logistic regression analysis of factors affecting PMV in COVID-19 patients with IFI (*n* = 150).

Parameter	Category (Description)	Univariate		Multivariate	
		OR (95%CI)	*p*-Value	aOR (95%CI)	*p*-Value
BMI	Obesity vs. non-obesity	2.218 (1.148–4.285)	0.018	1.478 (0.390–5.601)	0.565
COVID-19 Vaccination	Yes vs. no	0.370 (0.164–0.833)	0.014	0.155 (0.029–0.835)	0.030
Duration of corticosteroid treatment	1 day Increment	1.049 (1.011–1.088)	0.011	1.056 (0.978–1.140)	0.167
Neutrophils (%)	1% Increment	0.961 (0.927–0.996)	0.031	0.924 (0.826–1.035)	0.171
Protein (g/L)	1 g/L Increment	0.925 (0.859–0.995)	0.037	0.900 (0.819–0.989)	0.028
CRP (mg/L)	1 mg/l Increment	0.994 (0.989–0.999)	0.016	0.992 (0.981–1.003)	0.154
PT (%)	1% Increment	1.025 (1.003–1.048)	0.029	0.944 (0.885–1.008)	0.084

Abbreviations: aOR, adjusted odds ratio; 95% CI, 95% confidence interval; BMI, body mass index; CRP, C-reactive protein; PT, prothrombin time.

**Table 4:** Cox regression analysis of factors affecting 30-day mortality in COVID-19 patients with IFI (*n* = 150).

Parameter	Category (Description)	Univariate		Multivariate	
		HR (95%CI)	*p*-Value	HR (95%CI)	*p*-Value
Age (years)	>60 vs. ≤60	2.161 (1.212–3.855)	0.009	1.765 (0.775–4.023)	0.176
CKD	Yes vs. no	2.287 (1.201–4.321)	0.012	1.177 (0.214–6.469)	0.851
Hypertension	Yes vs. no	2.048 (1.272–3.300)	0.003	1.626 (0.801–3.299)	0.179
History of Stroke	Yes vs. no	2.398 (1.250–4.600)	0.008	1.318 (0.461–3.764)	0.607
COVID-19 Vaccination	Yes vs. no	0.509 (0.315–0.822)	0.006	0.475 (0.219–1.029)	0.059
WBC (×10^9^/L)	1 × 10^9^/L Increment	1.061 (1.021–1.103)	0.003	1.011 (0.944–1.083)	0.756
Urea (mmol/L)	1 mmol/L Increment	1.042 (1.022–1.063)	<0.001	1.032 (0.979–1.087)	0.239
Glucose (mmol/L)	1 mmol/L Increment	1.003 (1.007–1.061)	0.014	1.047 (1.003–1.093)	0.036
CRP (mg/L)	1 mg/L Increment	1.005 (1.002–1.007)	<0.001	1.002 (0.998–1.006)	0.335
INR	1 unit Increment	1.955 (1.235–3.096)	0.004	1.623 (0.527–4.997)	0.399
NLR	1 unit Increment	1.015 (1.006–1.023)	0.001	1.024 (1.009–1.039)	0.002
Tracheostomy	Yes vs. no	0.337 (0.213–0.534)	<0.001	0.273 (0.127–0.589)	0.001
CRRT	Yes vs. no	0.336 (0.212–0.533)	<0.001	0.789 (0.385–1.617)	0.518

Abbreviations: HR, hazard ratio; 95%CI, 95% confidence interval; CKD, chronic kidney disease; WBC, white blood cells; CRP, C-reactive protein; INR, international normalized ratio; NLR, neutrophil-to-lymphocyte ratio; CRRT, continuous renal replacement therapy

## Data Availability

The data supporting the findings of this study can be obtained from the corresponding author upon reasonable request.

## Supplementary Materials

The following supporting information can be downloaded at: https://www.mdpi.com/article/10.3390/tropicalmed10050124/s1. Table S1: Laboratory parameters at IFI diagnosis of the study patients according to mortality, Table S2: Lesions on CT findings of the study patients (*n* = 79), Table S3: Antifungal treatment characteristics of the study patients (*n* = 150), Table S4: Univariate regression analysis of factors affecting PMV in COVID-19 patients with IFI (*n* = 150), Table S5: Simple Cox regression analysis of factors affecting 30-day mortality in COVID-19 patients with IFI (*n* = 150). Table S6: Spearman correlations among independent continuous variables chosen from a simple Cox regression model.

## Abbreviations

AKI	Acute Kidney Injury
BMI	Body Mass Index
CAIFIs	COVID-19-associated Invasive Fungal Infections
CAM	COVID-19-associated Mucormycosis
CAPA	COVID-19-associated Pulmonary Aspergillosis
CCA	Chromogenic Candida Agar
CKD	Chronic Kidney Disease
COPD	Chronic Obstructive Pulmonary Disease
COVID-19	Corona Virus Disease 2019
CRP	C-Reactive Protein
CT	Computed Tomography
ECMO	Extracorporeal Membrane Oxygenation
ETA	Endotracheal Aspirate
IFIs	Invasive Fungal Infections
ICUs	Intensive Care Units
IMV	Intensive Mechanical Ventilation
LMICs	Low- and middle-income Countries
PMV	Prolonged Mechanical Ventilation
CRRT	Continuous Renal Replacement Therapy
RT-PCR	Reverse Transcription Polymerase Chain Reaction
SARS-CoV-2	Severe Acute Respiratory Syndrome Coronavirus 2
SDA	Sabouraud Dextrose Agar
VAP	Ventilator-associated Pneumonia
