# Supplementary material for: Factors Associated with Prolonged Mechanical Ventilation and 30-Day Mortality in Intubated COVID-19 Patients with Invasive Fungal Infections: A Retrospective Observational Study
Source: Trop Med Infect Dis. 2025 May 6;10(5):124. doi: 10.3390/tropicalmed10050124 (PMC12115372; doi:10.3390/tropicalmed10050124)
Supplement: Supplementary file 1 [file tropicalmed-10-00124-s001.zip › tropicalmed-3594999-supplementary.pdf]

Supplementary data for the following study:

Factors Associated with Prolonged Mechanical Ventilation and 30-Day Mortality in Intubated COVID-19 Patients with Invasive Fungal Infections: A Retrospective Observational Study

Supplementary Table S1: Laboratory parameters at IFI diagnosis of the study patients according to mortality.

Parameter	Treatment outcome		*p*-value
	Discharged (*n*=54)	Death (*n*=96)	
WBC (×10^9/L)	9.5 (7.3-13.1)	11.2 (6.9-15.1)	0.290
Neutrophils (%)	87.7 (83.9-91.3)	90.1 (84.3-94.1)	0.018
Lymphocytes (%)	6.95 (4.18-10.55)	5.15 (2.43-9.48)	0.032
Hemoglobin (g/L)	125.0 (107.8-141.0)	120.0 (109.0-137.8)	0.472
Platelets (×10^9/L)	189.0 (159.8-239.8)	184.0 (145.0-243.5)	0.729
Urea (mmol/L)	5.7 (3.9-8.3)	8.4 (5.9-14.3)	<0.001
Creatinine (μmol/L)	72.0 (58.0-88.0)	82.9 (62.5-125.5)	0.008
Glucose (mmol/L)	8.35 (5.70-11.80)	10.60 (8.25-16.0)	0.002
Protein (g/L)	60.93 (7.41)	63.16 (8.30)	0.292
Albumin (g/L)	30.77 (6.28)	29.74 (5.30)	0.325
AST(UI/L)	54.0 (38.5-79.1)	46.4 (30.6-74.5)	0.143
ALT(UI/L)	35.1 (22.9-53.1)	33.0 (21.8-47.6)	0.425
CRP (mg/L)	70.9 (36.8-101.2)	90.0 (53.2-141.3)	0.030
Sodium (mmol/L)	136.0 (133.0-140.3)	137.0 (133.8-140.0)	0.714
Potassium (mmol/L)	3.80 (3.43-4.26)	3.99 (3.70-4.40)	0.06
Chloride (mmol/L)	101.0 (97.9-105.8)	101.5 (97.7-105.7)	0.692
Procalcitonin(ng/mL)	0.25 (0.13-0.53)	0.44 (0.17-1.30)	0.040
D-Dimer (ng/L)	1030 (576-3459)	1506 (884-4582)	0.047
PT (%)	78.0 (13.8)	74.6 (17.0)	0.209
APTT (s)	34.0 (29.6-39.2)	31.7 (28.8-36.2)	0.049
INR	1.16 (1.12-1.30)	1.24 (1.10-1.32)	0.172
Fibrinogen (g/L)	4.58 (1.13)	4.55 (1.17)	0.871
NLR	12.70 (8.47-19.85)	18.10 (8.98-39.04)	0.031
LCR	0.95 (0.48-1.86)	0.55 (0.33-1.24)	0.007

Normally distributed continuous data are demonstrated as mean and standard deviation (SD). Skewed continuous data are demonstrated as median and interquartile range (IQR).
WBC, white blood cells; AST, aspartate aminotransferase; ALT, alanine aminotransferase; CRP, C-reactive protein; PT, prothrombin time; APTT, activated partial thromboplastin time; INR, international normalized ratio; NLR, neutrophil-to-lymphocyte ratio; LCR, lymphocyte-to-C-reactive protein ratio

Supplementary Table S2: Lesions on CT findings of the study patients (*n*=79)

Characteristic	Probable CAPA (*n*=46)	Candidemia (*n*=33)
Ground glass opacities	43 (93.5)	29 (87.9)
Consolidation	35 (76.1)	30 (90.9)
Nodule	12 (26.1)	15 (45.5)
Cavity	5 (10.9)	5 (15.2)
Bronchial wall thickening	10 (21.7)	8 (24.2)
Pleural effusion	3 (6.5)	2 (6.1)
Interstitial infiltrates	1 (2.2)	0 (0)

Data are presented as *n*(%)

Supplementary Table S3: Antifungal treatment characteristics of the study patients (*n*=150)

Antifungal medication	Total (*n*=150)	Probable CAPA (*n*=46)	Candidemia (*n*=104)
Initial antifungal medication			
No antifungal therapy	28 (18.7)	6 (13.0)	22 (21.2)
Fluconazole	54 (36.0)	2 (4.3)	52 (50.0)
Itraconazole	5 (3.2)	5 (10.9)	0 (0)
Echinocandin	25 (16.7)	12 (26.1)	13 (12.5)
Voriconazole	37 (24.7)	20 (43.5)	17 (16.3)
Amphotericin B	1 (0.7)	1 (2.2)	0 (0)
Switched antifungal medication (n=51)			
Fluconazole	11 (7.3)	0 (0)	11 (10.6)
Itraconazole	1 (0.7)	1 (2.2)	0 (0)
Echinocandin	16 (10.7)	11 (23.9)	5 (4.8)
Voriconazole	21 (14.0)	13 (28.3)	8 (7.7)
Amphotericin B	2 (1.3)	0 (0)	2 (1.9)
Duration of antifungal treatment (days)	15 (8-29)	24 (15-36)	9 (7-15)

Data are presented as *n*(%) or median(IQR)

Supplementary Table S4: Univariate regression analysis of factors affecting PMV in COVID-19 patients with IFI (*n*=150)

Parameter	Category (description)	OR (95%CI)	*p*-value
Age	>60 vs ≤60 years	1.244 (0.605-2.557)	0.552
Gender	Male vs female	1.216 (0.637-2.323)	0.553
Diabetes	Yes vs no	1.574 (0.729-3.398)	0.248
Hypertension	Yes vs no	1.796 (0.931-3.465)	0.081
Malignancy	Yes vs no	1.328 (0.319-5.522)	0.697
SOFA score	1 score increment	1.041 (0.918-1.182)	0.529
Antifungal therapy	Yes vs no	0.558 (0.234-1.332)	0.189
Antiviral medication	Remdesivir vs no	0.340 (0.162-1.117)	0.056
WBC ($\times 10^9$/L)	1 $\times 10^9$/L increment	0.980 (0.922-1.041)	0.509
Lymphocytes (%)	1 % increment	1.036 (0.987-1.088)	0.156
Platelets ($\times 10^9$/L)	1 $\times 10^9$/L increment	0.998 (0.994-1.003)	0.459
Urea (mmol/L)	1 mmol/l increment	0.978 (0.938-1.019)	0.294
Glucose (mmol/L)	1 mmol/L increment	0.988 (0.933-1.046)	0.680
Albumin (g/L)	1 g/L increment	0.978 (0.919-1.040)	0.479
Sodium (mmol/L)	1 mmol/L increment	1.004 (0.959-1.051)	0.870
Potassium (mmol/L)	1 mmol/L increment	0.909 (0.566-1.458)	0.691
Chloride (mmol/L)	1 mmol/L increment	1.007 (0.965-1.052)	0.739
Procalcitonin (ng/mL)	1 ng/mL increment	1.005 (0.970-1.041)	0.778
APTT (s)	1 s increment	0.994 (0.974-1.016)	0.606
INR	1 unit increment	0.319 (0.074-1.384)	0.127
Fibrinogen (g/L)	1 g/L increment	0.830 (0.615-1.121)	0.224
NLR	1 unit increment	0.995 (0.981-1.009)	0.477
LCR	1 unit increment	1.012 (0.972-1.053)	0.568

OR, odds ratio; 95% CI, 95% confidence interval; SOFA, sequential organ failure assessment; WBC, white blood cells; APTT, activated partial thromboplastin time; INR, international normalized ratio; NLR, neutrophil-to-lymphocyte ratio; LCR, lymphocyte-to-C-reactive protein ratio

Supplementary Table S5: Simple Cox regression analysis of factors affecting 30-day mortality in COVID-19 patients with IFI (*n*=150)

Parameter	Category (description)	HR (95%CI)	*p*-value
Gender	Male vs female	1.200 (0.768-1.873)	0.423
Diabetes	Yes vs no	1.408 (0.859-2.307)	0.174
Malignancy	Yes vs no	1.765 (0.811-3.843)	0.152
BMI	Obesity vs non-obesity	1.100 (0.704-1.718)	0.676
SOFA score	1 score increment	1.064 (0.972-1.163)	0.177
Antifungal therapy	Yes vs no	0.859 (0.482-1.533)	0.607
Antiviral medication	Remdesivir vs no	0.588 (0.370-1.934)	0.250
Duration of corticosteroid treatment	1 day increment	0.965 (0.940-1.990)	0.070
ECMO	Yes vs no	0.317 (0.100-1.007)	0.053
Neutrophils (%)	1 % increment	1.045 (1.014-1.077)	**0.004**
Lymphocytes (%)	1 % increment	0.940 (0.900-0.982)	**0.005**
Platelets ($\times 10^9$/L)	1 $\times 10^9$/L increment	0.998 (0.990-1.003)	0.892
Albumin (g/L)	1 g/L increment	0.980 (0.941-1.021)	0.327
Protein (g/L)	1 g/L increment	1.034 (0.984-1.087)	0.186
Sodium (mmol/L)	1 mmol/L increment	1.033 (0.997-1.070)	0.076
Potassium (mmol/L)	1 mmol/L increment	1.180 (0.876-1.589)	0.277
Chloride (mmol/L)	1 mmol/L increment	1.043 (0.989-1.076)	0.100
Procalcitonin (ng/mL)	1 ng/mL increment	1.010 (0.995-1.024)	0.184
PT (%)	1 % increment	0.980 (0.967-0.993)	**0.002**
APTT (s)	1 s increment	1.006 (0.993-1.018)	0.386
Fibrinogen (g/L)	1 g/L increment	1.052 (0.842-1.315)	0.656
LCR	1 unit increment	0.907 (0.815-1.010)	0.075

HR, Hazard ratio; 95 %CI, 95% confidence interval; BMI, body mass index; SOFA, sequential organ failure assessment; ECMO, extracorporeal membrane oxygenation; PT, prothrombin time; APTT, activated partial thromboplastin time; LCR, lymphocyte-to-C-reactive protein ratio.

Supplementary Table S6: **Spearman correlations among independent continuous variables chosen from a simple Cox regression model.**

Parameter	WBC	Neutrophils	Lymphocytes	Urea	Glucose	CRP	INR	PT	NLR
WBC	1	0.680***	-0.650 ***	0.321***	0.180	0.257**	0.155	-0.177*	0.544***
Neutrophils	0.680***	1	-0.918***	0.206*	0.434***	0.266**	0.198*	-0.207*	0.928***
Lymphocytes	-0.650***	-0.918***	1	-0.301***	-0.441***	-0.202*	-0.208*	0.223**	-0.999***
Urea	0.321***	0.206*	-0.301***	1	0.272**	0.095	-0.003	-0.031	0.297***
Glucose	0.180	0.434***	-0.441***	0.272**	1	0.252*	0.170	-0.182	0.441***
CRP	0.257**	0.266**	-0.202*	0.095	0.252*	1	0.439***	-0.460***	0.203*
INR	0.155	0.198*	-0.208*	-0.003	0.170	0.439***	1	-0.987***	0.207*
PT	-0.177*	-0.207*	0.223**	-0.031	-0.182	-0.460***	-0.987***	1	-0.223**
NLR	0.544***	0.928***	-0.999***	0.297***	0.441***	0.203*	0.207*	-0.223**	1

*p < 0.05, **p < 0.01, ***p < 0.001.

WBC, white blood cells; CRP, C-reactive protein; INR, international normalized ratio; PT, prothrombin time; NLR, neutrophil-to-lymphocyte ratio.
